# CT-based radiomics nomogram to predict response of advanced adenocarcinoma of esophagogastric junction to neoadjuvant chemotherapy

**DOI:** 10.1186/s13244-025-02103-5

**Published:** 2025-10-08

**Authors:** Chuan-qinyuan Zhou, Yue-su Wang, Wen-han Liao, Jing-ke Li, Xin-yi Liao, Yan Gui, Xiao-ming Zhang, Tian-wu Chen

**Affiliations:** 1 Department of Radiology, Affiliated Hospital of Southwest Medical University, 646000 Luzhou, Sichuan China; 2 Precision Imaging and Intelligent Analysis Key Laboratory of Luzhou, Southwest Medical University, 646000 Luzhou, Sichuan China; 3 Medical Imaging Key Laboratory of Sichuan Province, and Department of Radiology, Affiliated Hospital of North Sichuan Medical College, 637000 Nanchong, Sichuan China; 4 Department of Oncology, Affiliated Hospital of North Sichuan Medical College, 637000 Nanchong, Sichuan China; 5 Department of Radiology, The Second Affiliated Hospital of Chongqing Medical University, Yuzhong District, 400010 Chongqing, China

**Keywords:** Esophagogastric junction, Adenocarcinoma, Neoadjuvant chemotherapy, Radiomics, Computed tomography

## Abstract

**Objective:**

To establish and validate a CT-based radiomics model to predict the response of adenocarcinoma of the esophagogastric junction (AEG) to neoadjuvant chemotherapy (NAC).

**Methods:**

259 consecutive AEG patients, receiving 3 cycles of NAC with docetaxel, oxaliplatin and S-1, were retrospectively retrieved from two centers. Patients from center 1 were randomly divided into training (*n* = 139) and internal validation (*n* = 60) cohorts. Patients from center 2 were assigned to the external validation cohort (*n *= 60). In the training cohort, tumour-region-based radiomics features were selected, and a radiomics model was established to differentiate between patients with disease control and those with disease progression. Clinical factors were selected to develop a clinical model, and were incorporated with radiomics features to develop a radiomics-clinical model. Models’ predictive performance and calibration ability were assessed with the area under the ROC curve (AUC) and calibration curve analysis, respectively. Decision curve analysis was used to evaluate the net clinical benefit of the models.

**Results:**

The radiomics model was developed with 9 core radiomics features, the clinical model was established by incorporating gross tumor volume, cT stage and Siewert type, and the clinical-radiomics model was established and plotted the nomogram. The clinical-radiomics model obtained better performance than the clinical or radiomics model (AUCs: 0.903 vs. 0.824 or 0.823, 0.899 vs. 0.813 or 0.800, and 0.895 vs. 0.804 or 0.719) in training, internal validation and external validation sets, respectively. The clinical-radiomics model showed the best calibration ability and the highest net benefit.

**Conclusion:**

The clinical-radiomics model can well predict the response of AEG to NAC.

**Critical relevance statement:**

We provided a radiomics-clinical model to well predict the response of adenocarcinoma of the esophagogastric junction to neoadjuvant chemotherapy, which can help select appropriate patients to undergo chemotherapy, avoiding inappropriate patients from enduring toxic side-effects due to chemotherapy and delaying other treatments.

**Key Points:**

- A radiomics model for adenocarcinoma of the esophagogastric junction can predict the response to neoadjuvant chemotherapy.
- A combined model integrating clinical and radiomics features can improve predictive performance.
- The combined model is helpful for clinicians to develop individualized treatment regimens.

**Graphical Abstract:**

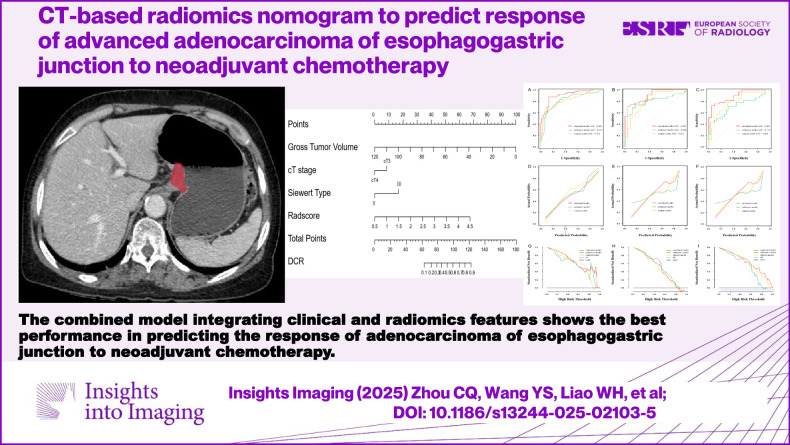

## Introduction

The adenocarcinoma of the esophagogastric junction (AEG) refers to the tumor center within 5 cm proximal or distal to the esophagogastric junction (EGJ), which involves the EGJ, and the incidence has increased rapidly in recent decades [1, 2]. Unfortunately, most AEG patients are diagnosed at an advanced stage. Neoadjuvant chemotherapy (NAC) has become the standard care for advanced AEG patients [3]. However, the response to NAC varies among patients due to the tumoral inherent heterogeneity, and patients with a poor response may have dismal survival [4]. Therefore, accurate prediction of the response to NAC is crucial, which can help develop individualized treatment for AEG patients.

Although histological tumor regression grade (TRG) remains the gold standard approach for response assessment in clinical settings, it is only obtainable after surgery, delaying the timely adjustment of therapy. Surgery is not routinely available for all patients. In fact, the Response Evaluation Criteria in Solid Tumors version 1.1 (RECIST 1.1) offers a widely accepted surrogate to guide therapeutic decisions [5, 6]. It allows for real-time monitoring of tumor dynamics without exposing patients to additional invasive procedures. Recently, radiomics has gained growing attention as a promising approach to extract mineable high-throughput quantitative features from medical images, which can reveal the potential biological characteristics and heterogeneity of tumor, providing information beyond the morphological and functional features [7–9]. Previous studies proved that radiomics represented a promising strategy for predicting complete response of gastric cancer and advanced AEG to NAC [10–14], demonstrating its promising predictive performance. Based on the previous publications, we hypothesize that radiomics may serve as a novel approach to predict the response of advanced AEG to NAC using docetaxel + oxaliplatin + S-1. To our knowledge, preoperative accurate prediction of the response of AEG to NAC using this treatment regime remains unexplored. Therefore, we aimed to develop and validate a CT-based radiomics nomogram to predict the response of advanced AEG patients to NAC for clinicians to develop individualized treatment regimens.

## Materials and methods

### Patients

Approval of this study was granted by the ethics committee of the Affiliated Hospital of North Sichuan Medical College (Approval No. 2023ER335-1). Written informed consent was waived due to the retrospective nature of this study.

From November 2017 to July 2023, the clinical and contrast-enhanced CT (CECT) imaging data of 269 consecutive patients with gastroscopically and histologically confirmed AEG at centers 1 and 2 were retrospectively retrieved. Portal venous-phase CT images were retrieved from the picture archiving and communication systems for further evaluation. The inclusion criteria were as follows: (1) pre-NAC clinical stage of cT_2_N_+_M_0_, (2) thoracoabdominal CECT performed one week before NAC and three days after 3 cycles of NAC, and (3) the intervals between the pre-NAC CT scan and the start of the therapy were less than two weeks. The exclusion criteria were as follows: (1) poor CT image quality and insufficient clinical data (*n* = 4); and (2) combined history of other malignancies (*n* = 6). Prior to NAC, positron-emission tomography was employed to determine whether there were metastases.

Finally, a total of 259 patients were enrolled on this study. 199 patients from center 1 were randomized and divided into the training cohort (*n* = 139) and the internal validation cohort (*n* = 60), and 60 patients from center 2 were assigned to the external validation cohort (*n* = 60). The details of all enrolled patients in the training, internal validation and external validation cohorts are displayed in Table 1.

**Table 1:** The baseline characteristics of the training, internal validation, and external validation cohorts

	Training cohort (*n* = 139)	Internal validation cohort (*n *= 60)	External validation cohort (*n* = 60)	*p*-value
Age	66.5 ± 3.9	66.5 ± 9.5	65.1 ± 4.6	0.978
Gender				0.821
Male	98 (70.5)	42 (70.0)	44 (73.3)	
Female	41 (29.5)	18 (30.0)	16 (26.7)	
cT stage				0.915
cT_3_	70 (50.4)	33 (55.0)	31 (51.7)	
cT_4_	69 (49.6)	27 (45.0)	29 (48.3)	
cN stage				0.894
cN_0_	12 (8.6)	8 (13.3)	9 (15.0)	
cN_1_	34 (24.5)	15 (25.0)	13 (21.7)	
cN_2_	62 (44.6)	24 (40.0)	23 (38.3)	
cN_3_	31 (22.3)	13 (21.7)	15 (25.0)	
Siewert type				0.926
II	78 (56.1)	38 (63.3)	36 (60.0)	
III	61 (43.9)	22 (36.7)	24 (40.0)	
GTV	47.2 ± 23.9	46.1 ± 26.7	45.4 ± 25.7	0.967
CEA	27.6 ± 10.3	29.1 ± 14.7	32.1 ± 15.3	0.718
CA19-9	35.2 ± 15.2	41.1 ± 20.3	39.8 ± 16.5	0.625

*GTV* gross tumor volume, *CEA* carcinoembryonic antigen, *CA19-9* carbohydrate antigen 19-9

### CT scanning

The patients in our study underwent a CT scan using two 64 multi-detector systems (LightSpeed VCT, GE Medical Systems, USA; and SOMATOM Definition AS + , Siemens Healthineers, Erlangen, Germany). Prior to CT examination, all patients consumed 800 to 1000 mL of water as an oral negative contrast agent. Patients were scanned in the supine position. After the unenhanced CT scan, the arterial phase and portal venous phase images were obtained 25 and 70 seconds after intravenous injection of 1.5 mL/kg nonionic contrast agent (Omnipaque, Iohexol, GE Healthcare, USA) at the rate of 3.0 mL/s with a pump injector (Vistron CT Injection System, Medrad, USA) and flushed with 20 mL saline. Each scan was performed within a breath-hold for 10 to 15 s to get good quality images. The scan coverage extended from the right diaphragmatic dome to the inferior border of both kidneys. The parameters of the two CT scanners were given as follows: tube voltage of 120 kV, tube current of 200 mA, detector collimation of 64 × 0.6 mm, matrix of 512 × 512 mm, slice thickness of 1 mm, rotation time of 0.5 s, and pitch of 0.9 for the GE scanner. The scanning parameters for the Siemens scanner were similar to those for the GE scanner, except for the rotation time of 0.3 s, and the pitch of 0.8. The window settings were configured with a window width of 400 HU and a window level of 40 HU.

### Neoadjuvant chemotherapy

The NAC protocol was as follows: docetaxel at 75 mg/m^2^ and oxaliplatin at 130 mg/m^2^ were administered intravenously on day 1. Based on the patient’s body surface, S-1 was administered orally respectively on day 1–14 (80, 100 and 120 mg/time in the case of body surface area < 1.25 m^2^, 1.25–1.5 m^2^ and ≥ 1.5 m^2^, respectively).

### Image-based treatment assessment

**Fig. 1:**
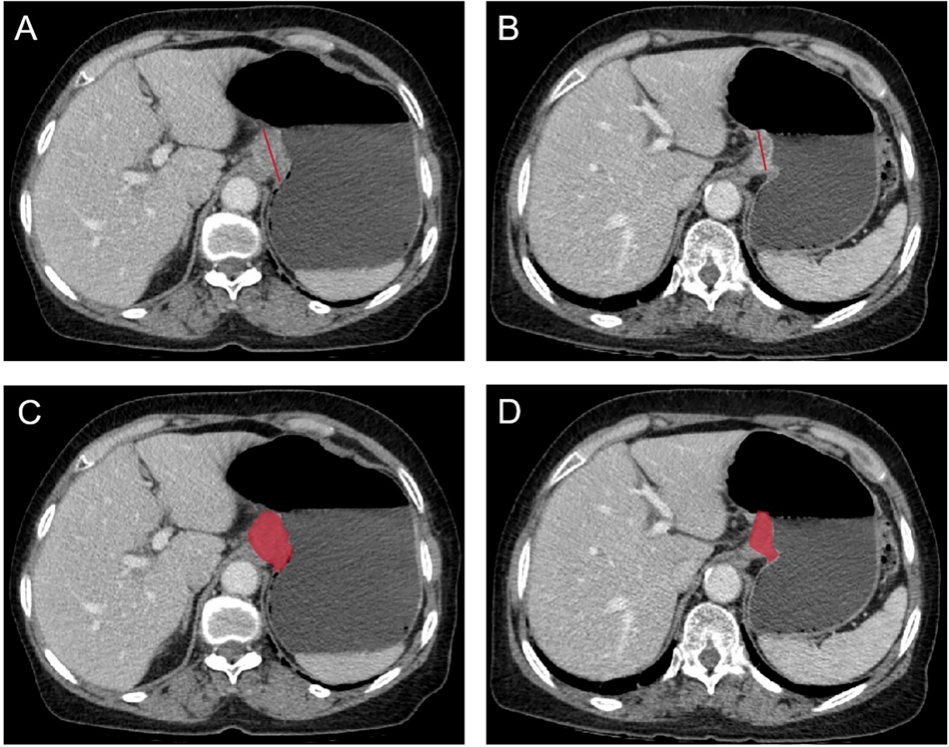
Advanced adenocarcinoma of esophagogastric junction in a 71-year-old man. **A**,** B** Show measurement of the tumoral longest diameter on portal venous phase axial CT image before and after neoadjuvant chemotherapy, respectively. **C**,** D** Depict segmentation of volume of interest on portal venous phase axial CT before and after neoadjuvant chemotherapy, respectively

The treatment response in all target lesions, including AEG and the positive lymph nodes, was assessed according to the RECIST 1.1. The treatment response was categorized as follows: (1) complete response (CR): disappearance of all target lesions; (2) partial response (PR): ≥ 30% decrease in the sum of diameters of target lesions (Fig. 1A, B), taking as reference baseline; (3) stable disease (SD): neither PR nor PD met; (4) progressive disease (PD): at least a 20% increase in the sum of diameters of target lesions, taking as reference the smallest sum on study. The index of disease control rate (DCR) included CR, PR and SD, and was used to evaluate the response to NAC using docetaxel, oxaliplatin and S-1.

### Interpreting of pre-NAC CT images

The pre-NAC cT and cN stages of AEG were evaluated on CECT according to the guidelines of the 8th edition of the American Joint Committee on Cancer (AJCC). The Siewert Classification was evaluated according to the distance from the tumor center to the EGJ. AEG was classified as: type I, epicenter 1–5 cm above the EGJ; type II, 1 cm above and 2 cm below the EGJ; and type III, epicenter 2–5 cm below the EGJ.

The measurement of gross tumor volume (GTV) of AEG was conducted using 3D-Slicer by delineating regions of interest according to the tumor area slice-by-slice, with efforts made to minimize inclusion of air within the esophageal and gastric lumen (Fig. 1C, D). The software automatically calculated the tumor volume.

### Pre-NAC image processing and VOI segmentation

The portal venous images were resampled to a voxel size of 1 × 1 × 1 mm to mitigate the potential impact of diverse scanning protocols or equipment on the extraction of radiomics features. The voxel intensity values were discretized using a fixed bin width of 25 HU to mitigate image noise and standardize intensities, thereby ensuring consistent intensity resolution across all tumor images. The volume of interest (VOI) was segmented by delineating around the tumor outline slice by slice on the 3D-slicer (Fig. 1C). Extensive precautions were exercised to exclude the gastric cavity, stomach contents, and fatty tissue around the stomach wall during the segmentation process.

To ensure the repeatability of the radiomics features’ extraction, two radiologists (radiologists 1 and 2, with 4 and 3 years of experience in abdominal imaging, respectively) segmented the VOI independently on the images. The VOI segmentation was repeated by radiologist 1 one month later. The interobserver agreement was assessed by comparing the radiomics features extracted by radiologist 1 for the first time with those by radiologist 2. The intraobserver agreement was assessed by comparing the radiomics features extracted twice by radiologist 1. Before the VOI segmentation, the two radiologists received guidance from a radiology professor with 27 years of experience in abdominal imaging. During the process of VOI segmentation, both radiologists were supervised by this radiology professor.

### Pre-NAC radiomics feature extraction

The radiomics features were extracted from the VOI using radiomics module in 3D-Slicer, and the discrimination of radiomics features was enhanced by applying the Wavelet filter and Laplace of Gaussian filter with different sigma values to the segmentation. A total of 1223 radiomics features were extracted from the tumor VOI, including first order statistics, shape, gray-level co-occurrence matrix (GLCM), gray-level run length matrix (GLRLM), gray-level size zone matrix (GLSZM), gray-level dependence matrix (GLDM), and neighboring gray tone difference matrix (NGTDM). The *Z*-score standardization method was applied for the radiomics feature standardization and normalization, and the radiomics features exhibiting interobserver and intraobserver intra-class correlation coefficient (ICC) values over 0.75 were selected for further analysis.

### Pre-NAC radiomics model establishment

The feature selection and predictive model establishment were initially conducted in the training cohort; the same workflow was subsequently applied to validate our model performance in the internal and external validation cohorts. Univariate analysis and least absolute shrinkage and selection operator (LASSO) were used to select the core radiomics features, while 10-fold cross-validation was applied to identify the optimal hyperparameters and optimize the feature count. Based on the core radiomics features, a radiomics model was established by logistic regression analysis, and the predicted probability of the radiomics model served as the Radscore for each feature.

### Pre-NAC clinical and combined model establishment

This study collected the baseline clinical characteristics, including age, gender, Siewert type, GTV, Carcinoembryonic Antigen (CEA), Carbohydrate Antigen 19-9 (CA19-9), and clinical T (cT) and N (cN) stages. Univariate analysis and binary logistic regression analysis were used to select the independent predictors. Subsequently, based on the established Radscore and clinical independent predictors, a clinical-radiomics combined model was established.

### Model evaluation

The performance of the models was assessed through the receiver operating characteristic curve (ROC) analysis to obtain the area under the ROC curve (AUC). The sensitivity, specificity, and accuracy were used to evaluate the discriminatory ability of the models. The calibration ability of the models was mainly tested by calibration curve analysis. To compare the AUCs between different models, DeLong’s test was applied (*p* < 0.05, indicating a statistical difference). The decision curve analysis was used to evaluate the net clinical benefit obtained by the models at different threshold probabilities.

### Statistical analysis

Statistical analysis was performed using the IBM SPSS statistical software (version 25.0 for Windows SPSS, Chicago, IL, USA) and R software (version 3.6.3, http://www.r-project.org). The continuous variables were presented as mean ± standard deviation. Categorical variables were presented as numbers with the corresponding percentages (*n*, %).

Comparisons of continuous variables between two cohorts were performed using an independent samples *t*-test or Mann–Whitney *U*-test. Categorical variables comparisons between two cohorts were performed using the Chi-square test or Fisher’s exact test. A two-sided *p*-value < 0.05 was considered statistically significant. The R packages, including “psych”, “glmnet”, “pROC”, “rmda”, “rms” and “Hmisc” were applied to the radiomics and combined models’ establishment.

## Results

### Patients’ characteristics

The baseline characteristics of the training, internal validation, and external validation cohorts are presented in Table 1. No significant differences in patients’ characteristics were observed among the three cohorts.

### Radiomics model for predicting DCR

**Fig. 2:**
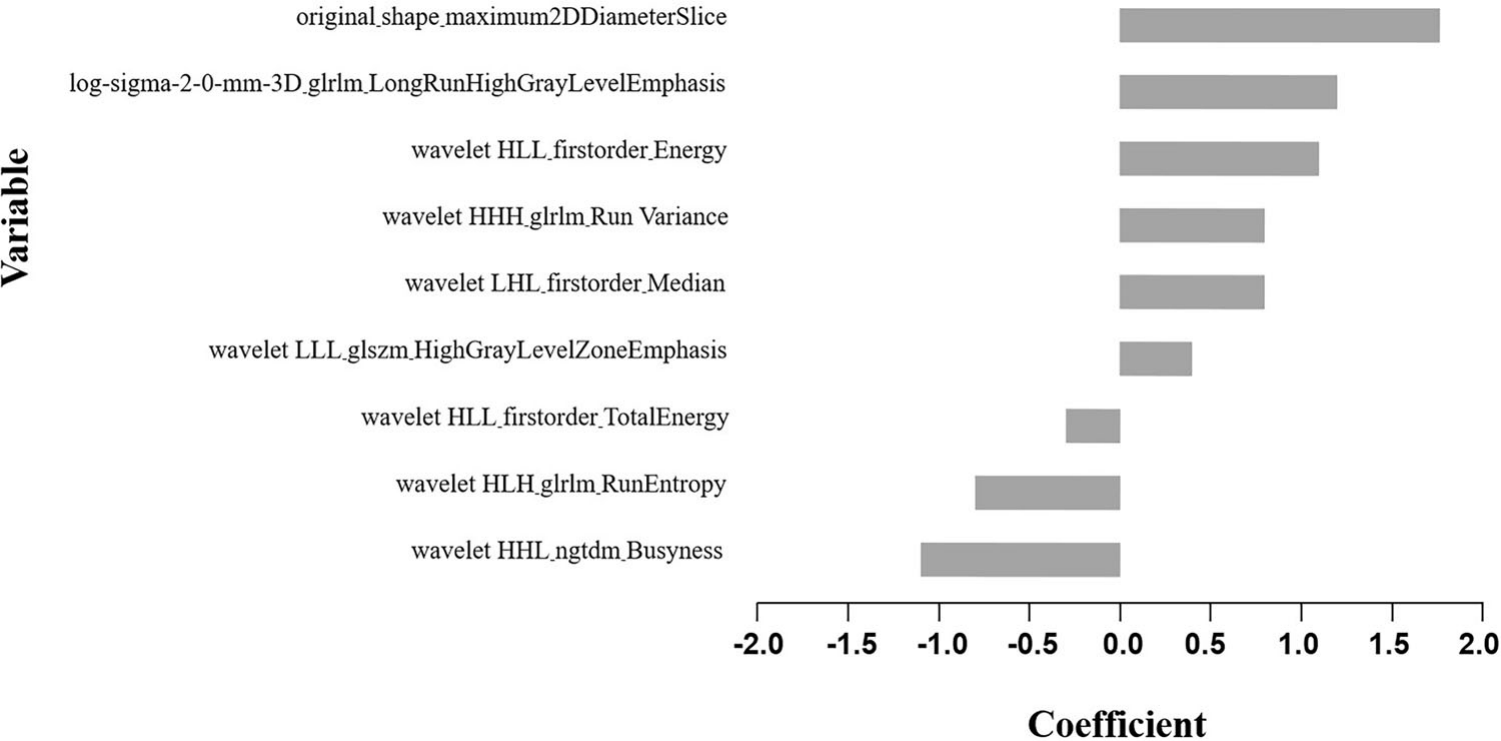
The 9 core radiomics features and their corresponding coefficients

In the training cohort, 1107 radiomics features with ICC > 0.75 were retained among the 1223 features through both interobserver and intraobserver ICC analysis. Subsequently, 9 core radiomics features were selected through the univariate analysis and LASSO, including 1 Shape feature, 3 First-order features, 3 GLRLM features, 1 GLSZM feature, and 1 NGTDM feature (Fig. 2). The radiomics model was established by the logistic regression analysis, and the predicted probability of the radiomics model served as the Radscore for each feature.

### Clinical model and combined model to predict DCR

**Fig. 3:**
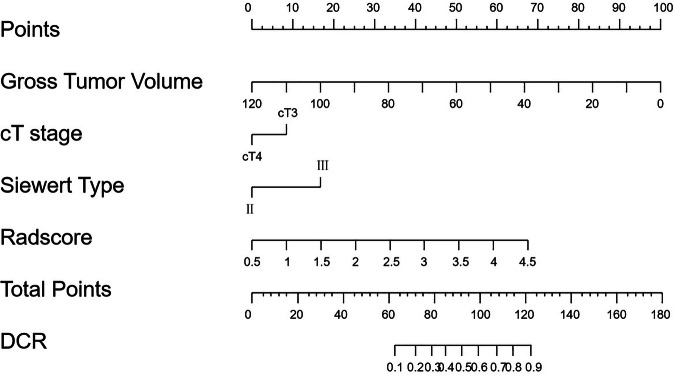
The nomogram of the clinical-radiomics combined model. DCR, disease control rate

In the training cohort, 100 patients achieved DCR, and 39 patients achieved PD. Subsequently, the univariate and binary logistic regression analysis revealed that GTV, cT stage, and Siewert type were independent predictors for DCR (Table 2), and the clinical model was established by incorporating the independent predictors. In comparison to AEGs classified as cT4, those classified as cT3 were more likely to achieve DCR. Similarly, AEGs classified as Siewert type III were more likely to achieve DCR compared to those classified as Siewert type II. Furthermore, AEGs with larger tumor volumes were associated with a decreased likelihood of achieving DCR. Based on the Radscore and clinical independent predictors, the combined model was established and plotted the nomogram (Fig. 3).

**Table 2:** Univariate and binary logistic regression analysis of independent predictors for the response to NAC in AEG patients

Factors	*β*	SE	Wald	*p*	OR	95% CI
cT stage	1.028	0.324	10.057	0.006	2.796	1.481–5.279
Siewert type	−1.157	0.321	12.977	0.002	0.314	0.157–0.590
GTV	−0.010	0.005	4.230	0.040	0.990	0.980–1.000

*NAC* neoadjuvant chemotherapy, *AEG* adenocarcinoma of esophagogastric junction, *GTV* gross tumor volume, *SE* standard error, *OR* odds ratio, *CI* confidence interval

### Models’ validation and comparisons

**Fig. 4:**
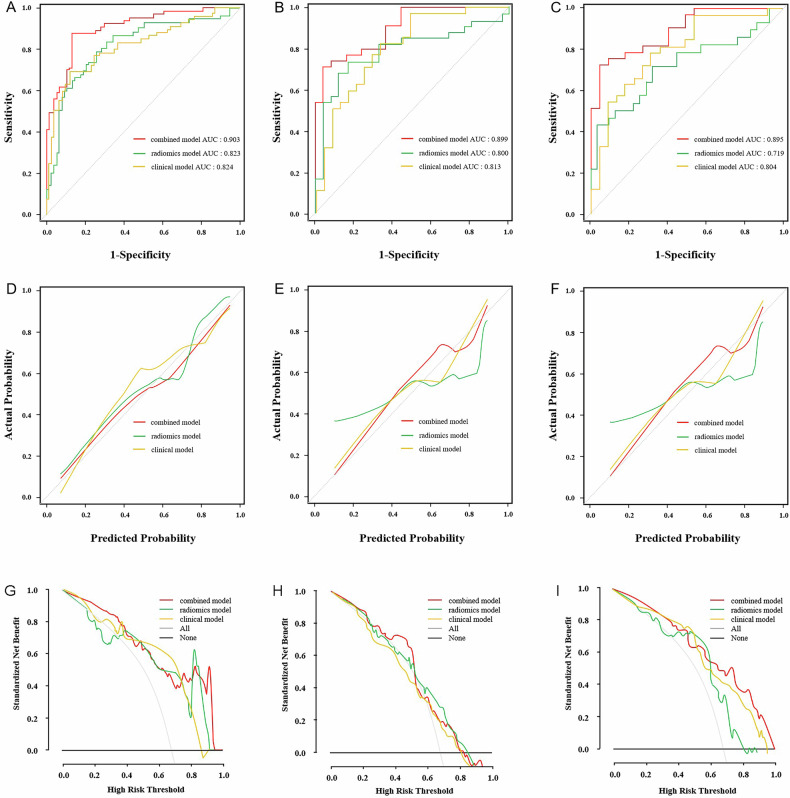
Performance of the radiomics, clinical and clinical-radiomics models. Images **A**, **B**, **C** show the ROC curves of the three models in the training, internal validation and external validation cohorts, respectively. Images **D**, **E**, **F** display the calibration curves of the three models in the training, internal validation and external validation cohorts, respectively. Among the calibration curves, the 45-degree sloping line indicates the ideal calibration. The closer to the ideal calibration line the model calibration curve, the greater the agreement between the model-predicted probability and the actual probability. Images **G**, **H**, **I** depict the decision curves of the three models in the training, internal validation and external validation cohorts, respectively. The larger the area under the curve for the same threshold probability interval, the higher the net benefit of the model. ROC, receiver operating characteristic curve

The AUC, specificity, and sensitivity of the radiomics models established for predicting DCR in the training, internal and external validation cohorts are shown in Table 3. The Delong’s test demonstrated a statistically significant difference in AUC between the combined model and either the clinical or radiomics model in the training cohort, with *p*-values of 0.021 or 0.002, respectively. However, there was no statistically significant difference in AUC between the clinical and radiomics model, with a *p*-value of 0.961. The combined model also had the highest AUC in the internal and external validation cohorts among the three models (Fig. 4A, B, C). The calibration curve analysis showed that the combined model had the best calibration ability in the training, internal validation and external validation cohorts among the three models (Fig. 4D, E, F). The decision curve analysis showed that the combined model had the highest net clinical benefit in the training, internal validation and external validation cohorts among the three models (Fig. 4G, H, I).

**Table 3:** Performance of models in the training, internal validation, and external validation cohorts for predicting the response to NAC

Factors	AUC	95% CI	Specificity	Sensitivity	Accuracy
Training cohort					
Clinical model	0.824	0.721–0.971	0.878	0.698	0.751
Radiomics model	0.823	0.764–0.963	0.743	0.794	0.759
Clinical-radiomics combined model	0.903	0.833–0.985	0.873	0.865	0.861
Internal validation cohort					
Clinical model	0.813	0.703–0.982	0.829	0.680	0.733
Radiomics model	0.800	0.697–0.973	0.743	0.840	0.766
Clinical-radiomics combined model	0.899	0.798–0.982	0.714	0.960	0.783
External validation cohort					
Clinical model	0.804	0.693–0.962	0.788	0.696	0.761
Radiomics model	0.719	0.721–0.986	0.714	0.688	0.683
Clinical-radiomics combined model	0.895	0.705–0.992	0.727	0.957	0.785

*NAC* neoadjuvant chemotherapy, *CI* confidence interval, *AUC* area under curve

## Discussion

Preoperative NAC has demonstrated promising outcomes in tumor downstaging and improving the rate of radical surgical resection [3, 15]. Despite the established efficacy of NAC in clinical practice, clinical evidence indicates that some patients exhibit resistance to this therapy and demonstrate a reduction in overall survival [4, 16, 17]. Therefore, it is of great significance to accurately identify AEG patients who are likely to respond to NAC prior to treatment to avoid participants with potential resistance receiving this therapy. In this retrospective study, we collected and analyzed the baseline clinical and enhanced CT features of AEG patients from two medical centers prior to NAC. Subsequently, we established the radiomics, clinical, and radiomics-clinical combined models that accurately predicted the response to NAC in AEG patients. Our findings indicated that the radiomics-clinical combined model had significant potential as a reference tool for predicting the response to NAC.

Radiomics systematically extracts the essential quantitative features from medical images, and the radiomics model is subsequently established, which represents a significant departure from traditional methods that rely on visual interpretation of images [7]. Liu et al [11] extracted the intratumoral and peritumoral radiomics features on the CECT images and established a combined radiomics-clinical model to effectively predict the pathological response to NAC in gastric cancer patients. Cui et al [18] performed a multicenter cohort study to predict the response to NAC in gastric cancer patients using the radiomics model. The model showed satisfactory discrimination of the response to NAC, and decision curve analysis confirmed its clinical utility, suggesting that it may be employed to identify optimal therapeutic strategies. However, there are limited reports employing radiomics to predict the response to NAC in AEG patients. Our study aimed to establish and validate a CT-based radiomics model to predict the response to NAC in AEG patients. We selected 9 core radiomics features on CT images, including 1 Shape feature, 3 Firstorder features, 3 GLRLM features, 1 GLSZM feature and 1 NGTDM feature. In the DCR group, the GLSZM feature had the lowest values. GLSZM described a homogeneous region within the tumor volume and the heterogeneity of the tumor on a regional scale. The lower GLSZM feature value of the lesion is, the more uniform the grayscale distribution of the lesion will be, suggesting a higher possibility of achieving DCR. Our finding can be supported by the study, which shows that the GLSZM feature values are positively correlated with tumor heterogeneity [19]. In this study, the radiomics feature extraction was performed on the portal venous phase CT images. Previous study demonstrated that the peak enhancement of tumors was significantly higher in the portal venous phase compared to the arterial phase, indicating a greater contrast between tumors and surrounding tissues [20]. Huang et al [14] established a combined radiomics-clinical model to predict the response to NAC in AEG patients, and pointed out that the AUC values of the radiomics model obtained using the portal venous CT images were superior to those obtained using the arterial CT images.

Among all the clinical features analyzed, the Siewert type, GTV and cTstage were related to the prediction of response to NAC, and the clinical model was established based on these clinical features to predict the response. Unlike the results of Huang et al [14], it was interesting to note that the Siewert type served as an independent predictor of the response to NAC in our study. Compared with Siewert type II AEGs, the background mucosa of Siewert type III AEGs exhibits a low degree of hypoatrophy and intestinal metaplasia. Research demonstrates that AEG with low degree hypoatrophy and intestinal metaplasia is less aggressive and has a better prognosis than AEG with marked degree hypoatrophy and intestinal metaplasia [21–24]. This may be the potential reasons for the Siewert type serving as an independent predictor of the response to NAC in our study.

The combination of radiomics and clinical features has improved the performance of the model in comparison with individual models. The potential reasons for the best performance of the combined model in this study may be related to the intratumoral heterogeneity. Studies have indicated that the radiomics and clinical features reflect the intratumoral heterogeneity and describe the biological behavior of the tumor, which are critical factors influencing the efficacy of NAC [25]. Studies have indicated that tumors with greater heterogeneity tend to be more aggressive in terms of metastasis and angiogenesis, and may demonstrate increased resistance to NAC [26, 27].

The study had some limitations. First, this study is a retrospective study. In the future, we will conduct a prospective study to confirm our findings. Second, this study only collected clinical and CT imaging data from patients. While the constructed model demonstrates satisfactory performance, it may remain limited by the absence of other pertinent data, such as pathological and immunological data. In the future, we will gather more data from patients, such as pathological and immunological data, to further improve the model performance.

In conclusion, our findings indicate that radiomics has significant potential as a reference tool for predicting the response to NAC in AEG patients prior to the treatment, and the combination of clinical and radiomics features significantly improves the performance of the model. Our radiomics-clinical model can help select appropriate patients to undergo NAC, avoiding inappropriate patients from enduring the toxic side effects due to chemotherapy and delaying other treatments.

## Data Availability

The dataset used or analyzed during the current study is available from the corresponding author on reasonable request.

## Abbreviations

AEG: Adenocarcinoma of esophagogastric junction
AUC: Area under the ROC curve
CA19-9: Carbohydrate antigen 19-9
CEA: Carcinoembryonic antigen
CECT: Contrast-enhanced CT
CR: Complete response
DCR: Disease control rate
EGJ: Esophagogastric junction
GLRLM: Gray-level run length matrix
GLSZM: Gray-level size zone matrix
GTV: Gross tumor volume
ICC: Intra-class correlation coefficient
NAC: Neoadjuvant chemotherapy
NGTDM: Neighboring gray tone difference matrix
PD: Progressive disease
PR: Partial response
RECIST 1.1: Response Evaluation Criteria in Solid Tumors Version 1.1
ROC: Receiver operating characteristic curve
SD: Stable disease
VOI: Volume of interest
